# Tuberculosis burden on AIDS in Brazil: A study using linked databases

**DOI:** 10.1371/journal.pone.0207859

**Published:** 2018-11-21

**Authors:** Valeria Saraceni, Adele Schwartz Benzaken, Gerson Fernando Mendes Pereira, Kleydson Bonfim Andrade, Patricia Bartholomay Oliveira, Denise Arakaki-Sanchez, Alessandro Caruso, Flavia Moreno Alves de Souza

**Affiliations:** 1 Health Situation Analysis Branch, Rio de Janeiro City Health Secretariat, Rio de Janeiro, Brazil; 2 Ministry of Health, Department of STIs, Aids and Viral Hepatites, Brasília, Brazil; 3 Ministry of Health, National Tuberculosis Control Program, Brasilia, Brazil; Agencia de Salut Publica de Barcelona, SPAIN

## Abstract

**Objectives:**

To estimate the burden of tuberculosis (TB) in reported AIDS cases, to compare the characteristics of TB/HIV subjects with those without TB and to evaluate survival with or without TB in Brazil.

**Methods:**

The data source was the linked database between AIDS (2011–2014) and TB (2011–2014) databases from the Notifiable Diseases Information System (SINAN). The sociodemographic, clinical, laboratory results and use of antiretroviral therapy (ART) data were compared by TB occurrence or not. Survival probability was estimated using the Kaplan-Meier method and associated factors were sought using Cox regression.

**Results:**

The proportion of TB diagnosed from 2011 to 2014 among AIDS cases reported between 2006 and 2014 was 6.3%. Subjects coinfected with TB were predominantly male, older, with lower schooling, with lower CD4 count, higher viral load, and higher proportion of ART initiation than those without TB. 57.5% were diagnosed with HIV before TB, 38.2% as concurrent TB/HIV and 4.3% with TB before HIV. 16,466 reported TB cases were not found in the AIDS database, although registered as HIV-infected in the SINAN TB database between 2011 and 2014. Median survival for PLHIV was 581 days, with 582 for those without TB, significantly higher than 547 for those with TB (log-rank teste, p = 0,001). In the Cox multivariate analysis, male gender [aHR = 1.27 (CI 95% 1.22–1.33)], older age [aHR = 1.020 (CI 95% 1.019–1.022)] and TB coinfection [aHR = 1.97 (CI 95% 1.88–2.07)] were positively associated with adjusted hazard of death, whereas CD4 count 200–499 cells [aHR = 0.21 (CI 95% 0.20–0.22)] and receiving ART [aHR = 0.2 2(CI 95% 0.21–0.23)] reduced the risk of death.

**Conclusions:**

HIV-infected subjects should be screened for TB at care entry, to minimize diagnosis and treatment delays when active TB is present or to increase the odds of being offered latent TB infection therapy to prevent TB. On the other hand, TB cases should be promptly tested for HIV. All those will contribute to reduce mortality among people living with AIDS.

## Introduction

People living with HIV/AIDS (PLHIV) present an increased risk of developing active tuberculosis (TB) [1]. In Brazil, the burden of TB in PLHIV has been measured through the HIV/TB coinfection cases included in the Notifiable Diseases Information System (SINAN). A study using this national database showed a coinfection incidence of 9.4% in the period between 2001 and 2011 [2]. The World Health Organization (WHO) estimated a TB/HIV coinfection prevalence around 13% (11 to 15%) in Brazil in 2015, ranking Brazil among the 30 countries with the highest burden of this double epidemics [3].

Considering the data obtained through the analysis of medical charts of 4,549 PLHIV in the city of Rio de Janeiro, Brazil, the prevalence of active TB in this population was 10.5% in the period between 2005 and 2009 [4]. In most PLHIV cases, active TB results from latent TB infection, which can be diagnosed and treated, reducing the risk of TB development and, consequently, reducing the mortality rate [5]. Results can be significantly improved if patients start antiretroviral therapy (ART) before or during TB treatment, thus reducing the risk of death between 44 and 71% [6].

Both AIDS and TB are included in the third Sustainable Development Goal which aims to achieve the eradication of both epidemics by 2030 [3]. The indicator number 9 of the “Strategy to End Tuberculosis” reinforces the need to document HIV status in 100% of the population reported with TB. The global expanded access to ART has collaborated with the reduction of HIV/AIDS mortality rates, and the “Test and Treat” strategy, as a component of the prevention and control of the HIV epidemics, reinforces the need to start ART on PLHIV as quickly as possible [7].

In 2016, 76.3% of reported TB new cases in Brazil had been tested for HIV, indicating a coinfection incidence of 9.4% among these patients [8]. However, this estimate derived from TB cases only, not taking into account the number of PLHIV developing active TB. In 2015, data from the HIV care cascade in Brazil showed 827000 estimated PLHIV in Brazil, with 87% already diagnosed, 80% of these on ART, and 90% of these with undetectable HIV viral load [9], potentially at risk of developing TB.

The linkage of different AIDS and TB registries is a useful tool to qualify epidemiological surveillance data, since it can provide greater knowledge about the magnitude of TB and HIV/AIDS coinfection. Both diseases require mandatory reporting in Brazil and data are available in distinct information databases [10]. Besides SINAN, other administrative databases were used by the Department of Surveillance, Prevention and Control of STIs, HIV/AIDS and Viral Hepatitis (DIAHV) to perform annual linkage using the software Reclink [11] with SIM (Mortality Information System), SISCEL (Laboratory Tests Control Systems) and SICLOM (Medication Logistics Control System), resulting in a more complete AIDS database, including information regarding CD4+-cell count, HIV viral load and ART use.

The aims of this study were to estimate the burden of TB in reported AIDS cases, to compare the characteristics of TB/HIV subjects with those without TB and to evaluate survival with or without TB in Brazil, given the universal access to ART [12].

## Methods

This study used the data resulting from the probabilistic linkage between the AIDS qualified database and SINAN TB. The AIDS qualified database comprises all the notifications to SINAN AIDS and the records recovered from SIM, SISCEL, and SICLOM [10]. The AIDS data available covered the period between 1980 and 2014, but only data from 2011 to 2014 were included. TB notifications were related to AIDS cases if TB was diagnosed in the period between 2011 and 2014. It should be pointed out that until 2014, only AIDS cases that met the notification criteria were reported to SINAN. Since then, HIV notification has become mandatory [13].

SISCEL and SICLOM administrative databases were made available at different times: SISCEL database in 2000 and SICLOM in 2006. SICLOM database covers all PLHIV on ART, both in public and in private healthcare sectors. SISCEL database, on the other hand, covers only those PLHIV who had CD4 and viral load tests conducted in the public laboratory network.

Due to the greater difficulty of diagnosing pulmonary TB in individuals younger than 15 years of age, this study opted to analyze AIDS cases in patients aged 15 or more, at risk of developing TB, because they would be more easily diagnosed through sputum smear tests.

In order to compare notified TB cases with registered HIV positive results in SINAN-TB, which were not found in the AIDS database, with the TB/HIV cases reported on both databases, only sociodemographic and TB-related variables available in SINAN were used.

The complete AIDS database contains information about gender, age at diagnosis of HIV/AIDS, race/color, schooling, municipality and state of residence, date of HIV diagnosis, category of HIV exposure, date of death (if found in SIM database), date and results of CD4 count exam conducted closest to diagnosis date, date and value in copies of viral load exams conducted closest to diagnosis date, information regarding the use of ART, and the date that ART was first dispensed. Variables related to tuberculosis were collected from the TB database, such as entry date (new cases, relapse, retreatment after default, transfer between health units), type of TB (pulmonary or extra-pulmonary), positive or non-positive pulmonary TB (positive result of a sputum smear and/or sputum culture and/or rapid molecular testing), associated diabetes, associated alcoholism, and TB treatment outcome (cure, default, death, drug resistant TB or outcome not registered in SINAN TB).

The variable related to the date of HIV diagnosis was redefined among the date of HIV diagnosis in SINAN, the date of first ART dispensing (SICLOM), and the date of diagnosis registered in SISCEL, whatever occurred first. The year of diagnosis was derived from this date. The year relative to the diagnosis of TB was determined from the “date of diagnosis” variable in SINAN TB database.

Time in days between the beginning of ART and the diagnoses of TB was calculated as the difference between the date ART was first dispensed and the date TB treatment was initiated.

The difference in days between the diagnosis of TB and HIV was calculated as the difference between TB diagnosis date and the new variable related to the date of HIV diagnosis. Later, this time period was categorized in three, defined as diagnosis of TB in a previously diagnosed HIV positive patient (for those who were diagnosed with TB after learning about their HIV-infected status), concurrent TB/HIV (when the diagnosis of HIV happened at the same time as TB or up to 270 days after TB diagnosis), and TB diagnosed before HIV (when the diagnosis of TB occurred before the diagnosis of HIV).

For the survival analysis, the difference between the date of death–for those found in SIM database–and the date of HIV diagnosis provided the survival time, in days, resulted in of the survival time. Death was considered as failure at the date of death. The administrative censoring was applied for all cases who weren’t found in SIM up to December 31, 2014, the end of the period of observation for the study. Cases who were found in SIM and whose survival time were zero, due to date of HIV diagnosis equals to date of death, had their survival time converted to 1 to allow their inclusion in the analysis.

In order to analyze survival rates, the AIDS cases diagnosed in the period between 2011 and 2014 were selected, according to their availability of the SINAN TB database.

Frequency distribution and central tendency and dispersion measures were obtained and compared. For the categorical variables, Pearson’s chi-squared test was used, and for the continuous variables, Student’s T-test or Wilcoxon test was applied. The trend analysis was conducted using the chi-squared test.

Survival analysis followed the Kaplan-Meier method, with the log-rank test or the Peto test to evaluate differences in survival probabilities. In order to obtain potential associated factors, we used Cox’s technique of proportional risks. To enter the model, the variables had to present a p-value < 0.20 in the bivariate analysis. All tests had a significance level of 0.05.

Multivariate survival analysis was limited to cases that contained information about CD4 count, in order to allow the stratification of the risk by CD4-cell count.

All analyses were conducted in Stata SE v.12, with a 5% significance level.

The database linkage that gave rise to this study is part of the routine work of the both the National Tuberculosis Control Program and the DIAHV, which, through probabilistic linkage of databases, attempts to improve existing information to increase the quality of TB and AIDS epidemiological surveillance in Brazil. The linkage was conducted using the RECLINK III software [11]. The variables “name”, “mother’s name”, “gender” and “date of birth” were compared using a probabilistic record linkage technique. After the linkage a score is established and a visual comparison is done to determine the cut-off point for cases considered as true-pairs. Others variables can the incorporated in this step, in order to facilitate the decision. The linkage steps are repeated with those not considered true-pairs, using other combinations of the same variables, e.g., year of birth instead of date of birth or mother’s first name only instead of first and last.

The request to access the mentioned databases was done through the Citizen’s Electronic Services Information System (e-SIC) and, because they do not contain patient´s personal identification, this access did not require submission to and approval by a Research Ethics Committee. All the procedures were conducted in compliance with Resolutions numbers 466/12 and 510/2016 issued by the National Health Council.

## Results

Between 2011 and 2014 there were 245913 reported cases of AIDS in PLHIV older than 14 years old, with 15543 presenting an episode of TB between 2011 and 2014 (6.3%). The years of HIV and TB diagnosis coincided in 69.1% of the cases in 2011, 72.0% in 2012, 75.6% in 2013, and 85.1% in 2014, pointing to a trend of HIV diagnosis performed during TB treatment.

Comparing the TB/HIV cases to those without TB (Table 1), it was seen that the TB/HIV group had a higher proportion of male gender (74.3% versus 65.0%), higher median age (37 versus 34 years of age), higher proportion of people with less than 4 years of schooling (9.0% versus 4.6%), higher proportion of deaths (25.8% versus 10.3%), lower median T-CD4+ cell count (118 versus 207 cells/mm^3^), higher HIV viral load (50563 versus 18549 copies), and similar proportion of ART initiation (72.0 versus 772.7%).

**Table 1:** Characteristics of AIDS cases with or without TB diagnosis in the period between 2011 and 2014, Brazil.

Characteristics	AIDS (n = 230370)	AIDS with TB (n = 15543)	p-value
Gender (n,%)			
Female	80573 (35.0)	3997 (25.7)	
Male	149617 (65.0)	11542 (74.3)	<0.001
Age (median, IQR)	34 (27–44)	37 (30–45)	<0.001
Race/color			
White	56842 (24.7)	3871 (24.9)	
Black	12045 (5.2)	1438 (9.3)	
Asian	603 (0.3)	39 (0.2)	
Brown	47047 (20.4)	4973 (32.0)	
Indigenous	337 (0.1)	43 (0.3)	
Ignored	113496 (49.3)	5179 (33.3)	<0.001
Schooling			
Up to 4 years	10577 (4.6)	1397 (9.0)	
4-plus years	85730 (37.2)	6741 (43.4)	
Ignored	134063 (58.2)	7405 (47.6)	< 0.001
Macro-region			
North	18544 (8.1)	1672 (10.8)	
Northeast	40161 (17.4)	3466 (22.3)	
Southeast	89464 (38.8)	6063 (39.0)	
South	45155 (19.6)	3271 (21.0)	
Center West	14957 (6.5)	699 (4.5)	
Ignored	22089 (9.6)	372 (2.4)	< 0.001
Death notified to SIM			
No	206635 (89.7)	11541 (74.2)	
Yes	23735 (10.3)	4002 (25.8)	<0.001
HIV-exposure category			
Homo/bisexual	37341 (16.4)	1962 (12.6)	
Heterosexual	69309 (30.1)	6237 (40.2)	
Drugs	612 (0.2)	203 (1.3)	
Hemophilia/transfusion	31 (0.0)	3 (0.0)	
Occupational exposure to blood	11 (0.0)	0 (0.0)	
Perinatal	1007 (0.4)	52 (0.3)	
Ignored	121659 (52.8)	7086 (45.6)	< 0.001
CD4 count (median, IQR)	207 (89–295)	118 (47–223)	<0.001
Viral load (copies, median, IQR)	18549 (3499–79404)	50563 (7851–179273)	<0.001
ART dispensing			
No	62976 (27.3)	4356 (28.0)	
Yes	167394 (72.7)	11187 (72.0)	0.062
Time, in days, between ART and TB (median, IQR)	-	-28 (-90, 22)	-

Source: AIDS and TB linked database.

Of the total number of AIDS cases between 2011 and 2014, 72.6% received ART, without a significant trend in the proportion of PLHIV entering ART in the period (p = 0.705).

Concerning the timing to diagnosis of AIDS and TB, 8941 (57.5%) of the cases were classified as HIV before TB, 5934 (38.2%) as concurrent TB/HIV and 668 cases (4.3%) as TB before HIV. As can be seen in Table 2, the groups were not similar, with concurrent TB/HIV having the highest proportion of outcome as death, while those with HIV diagnosed before TB had the lowest median CD4 count, and highest median viral load. ART initiation was higher in the HIV before TB group (81.0%). Median time in days between ART initiation and TB diagnosis was 69 days for the concurrent TB/HIV group, -6 days for the HIV before TB group, and 615 days for the TB before HIV group.

**Table 2:** Characteristics of AIDS cases found with TB in the period between 2011 and 2014, per timing of diagnosis, Brazil.

Characteristics	TB/HIV (n = 5934)	HIV first (n = 8939)	TB first (n = 668)	p-value
Gender (n,%)				
Female	1462 (24.6)	2340 (36.2)	195 (29.2)	
Male	4470 (75.4)	6599 (73.8)	473 (70.8)	0.012
Age (median, IQR)	37 (30–46)	36 (30–44)	36 (30–44)	0.001
Race/color				
White	1156 (19.4)	2610 (29.2)	105 (15.7)	
Black	539 (9.1)	847 (9.5)	52 (7.8)	
Asian	10 (0.2)	29 (0.3)	0 (0.1)	
Brown	1595 (26.9)	3229 (36.1)	149 (22.3)	
Indigenous	16 (0.3)	26 (0.3)	1 (0.1)	
Ignored	2618 (44.1)	2200 (24.6)	361 (54.0)	<0.001
Schooling				
Up to 4 years	535 (9.0)	823 (9.2)	39 (5.8)	
4-plus years	2061 (34.7)	4490 (50.2)	190 (28.4)	
Ignored	3338 (56.3)	3628 (40.6)	439 (65.8)	< 0.001
Macro-region				
North	520 (8.8)	1086 (12.1)	66 (9.9)	
Northeast	1405 (23.7)	1925 (21.5)	136 (20.4)	
Southeast	2392 (40.3)	3411 (38.2)	260 (38.9)	
South	1186 (20.0)	1936 (21.7)	149 (22.3)	
Center West	216 (3.6)	462 (5.2)	21 (3.1)	
Ignored	215 (3.6)	121 (1.3)	36 (5.4)	< 0.001
Death notified to SIM				
No	4251(71.6)	6749 (75.5)	541 (81.0)	
Yes	1683 (28.4)	2192 (24.5)	127 (19.0)	<0.001
HIV-exposure category				
Homo/bisexual	468 (7.9)	1440 (16.1)	54 (8.1)	
Heterosexual	1978 (33.3)	4085 (45.7)	174 (26.1)	
Drugs	86 (1.4)	108 (1.2)	9 (1.4)	
Hemophilia/transfusion	2 (0.0)	1 (0.0)	0 (0.0)	
Occupational exposure to blood	0 (0.0)	0 (0.0)	0 (0.0)	
Perinatal	15 (0.3)	36 (0.4)	1 (0.0)	
Ignored	3385 (57.1)	3271 (36.6)	430 (64.4)	< 0.001
CD4 Count (median, IQR)	129 (52–229)	109 (44–216)	206 (105–299)	0.001
Viral load (copies, median, IQR)	50597 (6059–181130)	52815 (9446–182737)	29839 (4202–124034)	0.001
ART initiated				
No	2066 (34.8)	2044 (22.9)	246 (36.8)	
Yes	3868 (65.2)	6897 (77.1)	422 (63.2)	<0.001
Time, in days, between ART and TB (median, IQR)	69 (33, 147)	-6 (-93, 37)	615 (438, 900)	0.001
Type of TB entry				
New case	5269 (88.8)	7951 (88.9)	536 (80.2)	
Relapse/ after loss of follow-up	398 (6.7)	687 (7.7)	111 (16.6)	
Transfer	267 (4.5)	303 (3.4)	21 (3.0)	<0.001
Type of TB				
Pulmonary	4898 (82.5)	6466 (72.3)	579 (86.7)	
Extra-pulmonary	1035 (17.5)	2469 (27.6)	89 (13.3)	<0.001
Positive pulmonary TB				
No	1904 (38.9)	3297 (51.0)	167 (28.8)	
Yes	2994 (61.1)	3169 (49.0)	412 (71.2)	<0.001
Associated diabetes	133 (2.2)	197 (2.2)	9 (1.3)	<0.001
Associated alcohol abuse	1298 (21.9)	1490 (16.7)	130 (19.5)	<0.001
Outcome				
Cure	2924 (49.3)	4473 (50.0)	421 (63.0)	
Default	732 (12.3)	1268 (14.2)	169 (25.3)	
Death	1478 (24.9)	2064 (23.1)	10 (1.5)	
DR-TB	30 (0.5)	40 (0.5)	5 (0.8)	
Unknown	770 (13.0)	1096 (12.2)	63 (9.2)	<0.001

Source: AIDS and TB linked database.

Regarding the variables related to TB, more subjects were reported new TB cases in concurrent TB/HIV, with the pulmonary form appearing in greater proportion in the TB before HIV group, who also presented the highest laboratory confirmation of TB through sputum smear tests. Diabetes as an associated disease was not higher than 2.2% in average, and alcoholism was higher in the simultaneous TB/HIV group (21.9%). The highest cure ratio occurred in the TB before HIV group (63.0%) and the highest proportion of deaths was reported in the concurrent TB/HIV group (24.9%). The percentage of cases closed as DR-TB was less than 1% in all three groups.

Table 3 shows a comparison of the 15543 AIDS cases found in the TB linkage between 2011 and 2014 with the 16466 TB cases not found in the AIDS database, although they had an HIV positive test result in the TB notification over the same period. Again, the groups were heterogeneous in the proportional distribution among the categorical variables. Median age had similar values. Among the TB cases that had not been reported to SINAN AIDS there were a higher proportions of black/brown (*pardo*) race/color (55.8%), schooling under 4 years (55.4%), TB retreatment (34.8%), pulmonary TB (81.1%), TB laboratory-confirmed (49.3%), diabetes (3.2%), alcoholism (20.7%) and default as treatment outcome (24.4%). This profile points to a more vulnerable population, whose cases were not reported to SINAN AIDS, despite the registered positive antibody test in SINAN TB.

**Table 3:** Characteristics of TB/HIV found in both SINAN AIDS and TB and of TB cases reported as HIV-infected but not found in SINAN AIDS, Brazil, 2011 to 2014.

Characteristics	AIDS with TB notified (n = 15543)	TB with HIV not notified (n = 16466)	p-value
Gender (n,%)			
Female	3997 (25.7)	4711 (28.6)	
Male	11542 (74.3)	11753 (71.4)	<0.001
Age (median, IQR)	37 (30–45)	37 (30–45)	0.126
Race/color			
White	3871 (24.9)	5809 (35.3)	
Black	6411 (41.3)	9184 (55.8)	
Asian	39 (0.2)	89 (0.5)	
Indigenous	43 (0.3)	66 (0.4)	
Ignored	5179 (33.3)	1318 (8.0)	<0.001
Schooling			
Up to 4 years	1397 (9.0)	2624 (15.9)	
4-plus years	6741 (43.4)	9121 (55.4)	
Ignored	7405 (47.6)	4721 (28.7)	<0.001
Macro-region			
North	1672 (10.8)	1202 (7.3)	
Northeast	3466 (22.3)	3703 (22.5)	
Southeast	6063 (39.0)	6737 (40.9)	
South	3271 (21.0)	4110 (25.0)	
Center West	699 (4.5)	626 (3.8)	
Ignored	372 (2.4)	88 (0.5)	<0.001
Type of TB entry			
New case	13756 (88.5)	10001 (60.7)	
Relapse	1196 (7.7)	5723 (34.8)	
Transfer	591 (3.8)	742 (4.5)	<0.001
TB type			
Pulmonary	11943 (76.9)	13354 (81.1)	
Extra-pulmonary	3593 (23.1)	3112 (18.9)	<0.001
Positive pulmonary TB			
No	8753 (56.3)	8345 (50.7)	
Yes	6790 (43.7)	8121 (49.3)	<0.001
Associated diabetes	339 (2.2)	523 (3.2)	<0.001
Associated alcoholism	2918 (19.1)	3409 (20.7)	<0.001
Outcome			
Cure	7818 (50.3)	7095 (43.1)	
Abandonment	2169 (13.9)	4024 (24.4)	
Death	3552 (22.9)	2655 (16.1)	
TB DR	75 (0.5)	141 (0.9)	
Not closed in SINAN	1929 (12.4)	2551 (15.5)	<0.001

Source: AIDS and TB linked database.

The survival analysis of the 245913 AIDS cases from 2011 to 2014, with or without associated TB, revealed that the mean survival time from the date of the AIDS diagnosis was 619 days, with a median of 581 days (IQR 216–995). When the subjects who did not have TB in the period of analysis were analyzed separately, the median survival time was 582 days (IQR: 219–995), significantly higher than the median of 547 days (IQR 146–981) for those who had TB (p = 0,001).

PLHIV who were diagnosed with HIV before TB had a higher survival (median: 642 days, IQR 223–1064), whereas those who had concurrent TB/HIV diagnosis had a lower one (median: 437 days, IQR 70–881). Finally, those with a TB diagnosis before AIDS had a median of 292 (IQR: 85–555) days.

**Fig 1:**
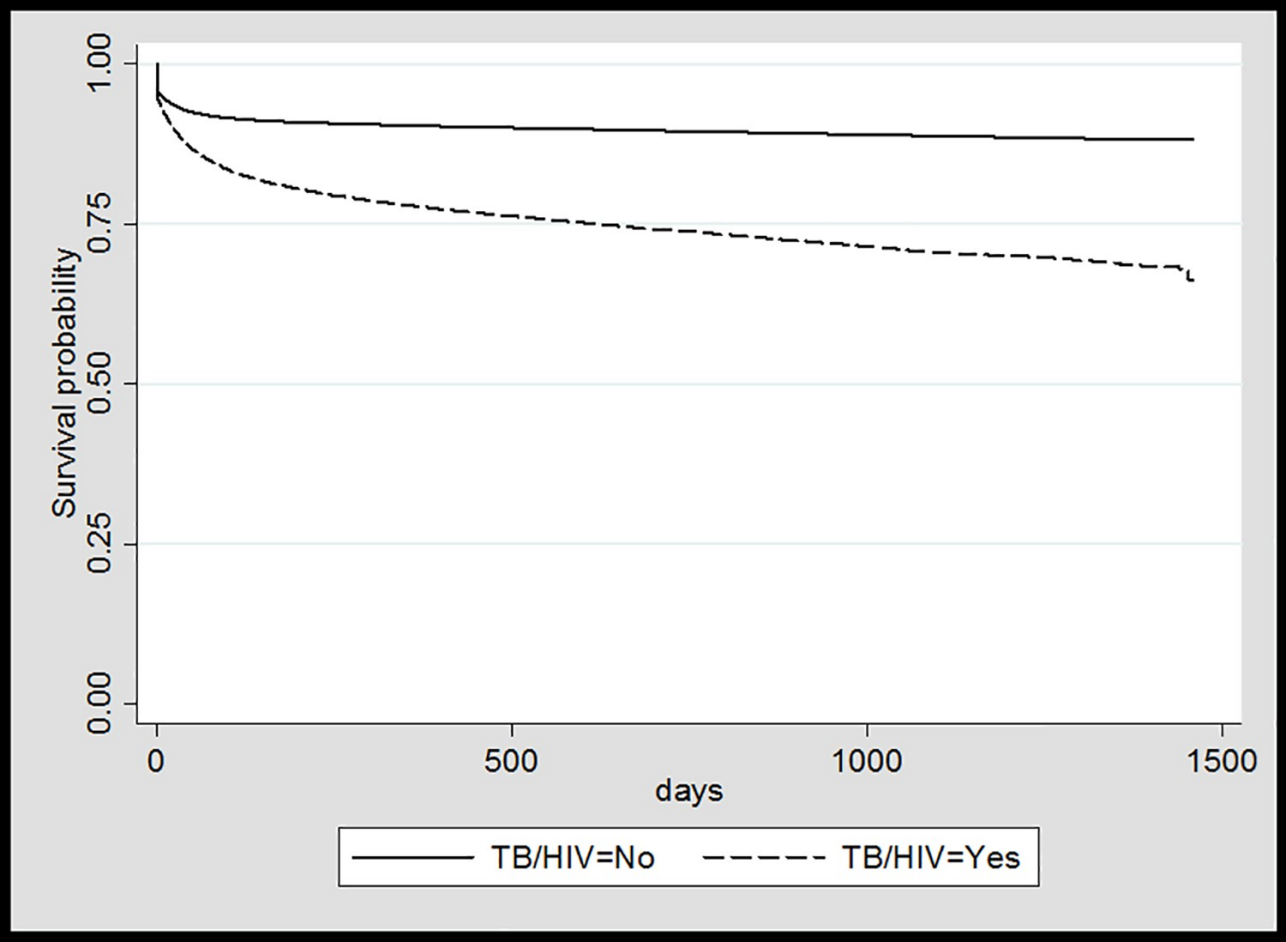
Probability of death in the AIDS cohort by TB coinfection, Brazil, 2011 to 2014. Source: AIDS and TB linked database.

The probability of survival was higher for those who were not diagnosed with TB in the period (log-rank test, p<0,001), as shown in Fig 1.

The factors associated with the risk of death were sought using Cox modelling (Table 4) for the 107874 individuals with CD4 count available in the period between 2011 and 2014. In the univariate analysis, the risk of death was positively associated with the male gender [HR = 1.39 (CI 95% 1.33–1.45)], with age [HR = 1.023 (CI 95% 1.022–1.025)] and with TB diagnosis [HR = 2.68 (CI 95% 2.56–2.81)]. On the other hand, the risk of death decreased substantially for the following categories: > = 4 years of schooling [HR = 0.63 (CI 95% 0.59–0.68)], white race/color [HR = 0.81 (CI 95% 0.77–0.85)], CD4 count between 200 and 499 cells [HR = 0.18 (CI 95% 0.17–0.19)] or CD4 count > = 500 cells [HR = 0.14 (CI 95% 0.10–0.20)] and starting ART [HR = 0.23 (CI 95% 0.22–0.24)]. In the multivariate analysis, male gender [aHR = 1.27 (CI 95% 1.22–1.33)], the increase of one year in age [aHR = 1.020 (CI 95% 1.019–1.022)] and coinfection with TB [aHR = 1.97 (CI 95% 1.88–2.07)] were associated positively with death, whereas CD4 count between 200 and 499 cells [aHR = 0.21 (CI 95% 0.20–0.22)] and ART initiation [aHR = 0.22 (CI 95% 0.21–0.23)] reduced the risk of death, remaining in the final model.

**Table 4:** Factors associated with death in the AIDS cohort registered in SISCEL, Brazil, 2011 to 2014.

Characteristics	Crude HR, CI 95%	Adjusted HR, CI 95%	p-value
Male	1.39 (1.33–1.45)	1.27 (1.22–1.33)	<0.001
Age (in years)	1.023 (1.022–1.025)	1.020 (1.019–1.022)	<0.001
Schooling > = 4 years	0.63 (0.59–0.68)	-	
White race/color	0.81 (0.77–0.85)	-	
CD4 count (cells/mm^3^)			
< 200	1 (Ref.)	1 (Ref.)	
200–499	0.18 (0.17–0.19)	0.21 (0.20–0.22)	<0.001
> = 500	0.14 (0.10–0.20)	-	
ART initiation	0.23 (0.22–0.24)	0.22 (0.21–0.23)	<0.001
TB coinfection	2.68 (2.56–2.81)	1.97 (1.88–2.07)	<0.001

Source: AIDS and TB linked database.

## Discussion

Among the AIDS cases reported from 2011 to 2014, 6.3% were diagnosed with a TB episode in the same period, whether concurrently to their HIV diagnosis or after having knowledge of their HIV+ status. Nevertheless, we found another 16,466 TB/HIV cases not reported as HIV-infected in the AIDS database, which would bring the proportion of TB/HIV coinfection closer to the WHO´s estimate of 13% [3].

Our finding of 6,3% accounts for just over half the TB prevalence found in Rio de Janeiro between 2005 and 2009 (10.5%), determined through the review of HIV-infected patients’ medical charts [4]. In Recife, the proportion of TB cases among PLHIV treated in referral centers was 7.1% [14]. Magno and colleagues linked the SINAN AIDS database of the state of Amazonas with SINAN TB in the period between 2001 and 2012, finding a proportion of concurrent TB/HIV or HIV before TB in 11.4% [15]. The smaller proportion found in this study may be a result of using secondary data, probably due to the previously demonstrated occurrence of underreporting of cases in SINAN TB [16] or the fact that a case had to meet the AIDS criteria to be reported by the time of diagnosis of a pulmonary TB [12].

Among the AIDS cases who had developed TB, 68.9% had their HIV diagnosis before the TB, with 77.1% starting ART a median of almost 1 week on ART by the time of TB diagnosis. The median time between ART and TB is compatible with studies that showed a TB diagnosis right after the initiation of ART, and relates it to the phenomenon of immune reconstitution [17]. On the other hand, those PLHIV may have benefited from TB latent infection diagnosis at the moment of HIV diagnosis, followed by treatment, a national recommendation [13] whose effectiveness is well recognized [5,18].

Other 38.2% were found as concurrent TB/HIV, probably because they were being offered the HIV test, as recommended in current protocols [8,10]. According to available data, 76% of all HIV/AIDS patients were started on ART in the period between 2006 and 2014, when the recommendations to initiate ART were in transition towards a universal indication [13].

TB/HIV subjects, when compared to those who did not develop TB, presented lower median CD4 count and higher median viral load. These results are compatible with a higher degree of immunosuppression and greater susceptibility to opportunistic infections. It is known that TB can be diagnosed in PLHIV regardless of a low CD4 count; however, TB prevalence increases in this population with the decrease of CD4 count [4, 19].

Regarding TB, those who were diagnosed with HIV before TB presented a higher proportion of extra-pulmonary TB, with a lower percentage of positive pulmonary TB and lower proportion of cure as treatment outcome in SINAN. A meta-analysis showed an association between HIV infection and extra-pulmonary TB, notably with lower levels of CD4 [20].

Between 2011 and 2014, 16466 other HIV-infected cases were reported in the SINAN TB, but weren’t found in the AIDS database. This fact may reflect underreporting in the SINAN AIDS in this period, maybe due to the fact that the individuals did not yet meet the criteria for AIDS notification. In the state of Amazonas, underreporting of AIDS based on TB notification with HIV-infected results was 19.6% [15]. In this study, 81% of TB cases with information of HIV-infected tests that were not registered in the AIDS database were pulmonary TB, which alone did not meet the criteria to be reported as AIDS cases. However, if the information regarding positive HIV test results was true, not only it would almost double the prevalence of TB/HIV in Brazil, but it would point out to a missed opportunity of including PLHIV in AIDS care and mostly, on ART, reducing the odds of death among them.

Several studies have reported the benefits of early ART initiation in TB/HIV co-infected subjects, with impact on mortality rates [21, 22]. The greater vulnerability of this group should draw the attention of healthcare professionals, regarding the inclusion and retention in healthcare services of PLHIV [9]. A study in Kenya has shown a 4-fold risk of death in PLHIV when compared to non-infected individuals, despite a 73.6% ART coverage [23]. In this study, 71% of reported PLHIV between 2006 and 2014 had started ART, with a significant tendency to increase the proportion of PLHIV receiving ART in the period.

Survival was significantly lower in the group with TB when compared to the group without TB in the period between 2011 and 2014, presenting a 2.7 times greater risk of death, which is in accordance with the current literature [24]. Among the factors that may change this reality are the introduction of ART at any moment after HIV diagnosis [5, 25], early start of ART after the diagnosis of TB [21, 22, 26], and the treatment of latent TB infection [18], all positively associated with improved prognosis and higher survival for PLHIV. This study showed that being registered to receive ART reduced the risk of death by 77%. A CD4 count above 200 cells at the moment of reporting also prompted a reduction of 82% in the risk of dying. In the Temprano trial, even PLHIV with high CD4-cell counts and on ART had a reduction in the risk of death after a course of LTBI treatment to prevent TB [27].

The linkage of DIAHV databases with SIM and SINAN TB allows a nationwide evaluation of TB coinfection without the need to collect primary data, and shows the importance of this methodology [11] in the analysis of the epidemiological situation in Brazil.

One of the limitations of this study was the fact that the linkage of the TB databases was limited to the period between 2011 and 2014, while the combined AIDS database was included in its totality, that is, AIDS cases could have presented with TB at some time prior to 2011. However, the gathered information built an interesting scenario about TB/HIV coinfection in Brazil, and may inform new strategies, some of which have already been presented as collaborative activities between healthcare programs [10] and, also the need to start ART on all PLHIV as soon as they are diagnosed [13].

Another limitation of this study was that it was not possible to include time-dependent variables as CD4 count, viral load and ART dispensing. However, the data used here has already provided a fresh perspective on the use of secondary data, a path not taken previously, by including data from administrative databases (SICLOM and SISCEL) without the need for medical chart reviews and allowing for more qualified analyses. The proportion of missing data in the databases that originated our dataset was another flaw, which happens with secondary data, and involved mainly sociodemographic data as years of schooling and race/color, limiting our proxy of socioeconomic status.

Although there is a great deal of information aggregated to the AIDS combined database, the lack of a registry for information on the diagnosis and treatment of latent tuberculosis infection has hindered an assessment of TB prevention among PLHIV and its impact on morbidity and mortality, as demonstrated in previous studies [5, 14].

The possible loss of HIV/AIDS cases notified with TB and not found in the AIDS database raises some issues. First, the importance of introducing a positive HIV test result as one of the criteria for AIDS notification, which occurred in 2014 in Brazil [12]. Second, the need to engage health surveillance teams on conducting an active search in their SINAN TB databases in order to investigate cases with HIV-infected result among TB notifications, which were not included in the HIV healthcare and in the SINAN AIDS. Finally, surveillance teams can make use of the variable “on ART”, available in the SINAN TB reporting form since 2014 to identify AIDS underreporting and go back to SICLOM to find them.

## Conclusions

Our study showed that the majority of TB/HIV cases were diagnosed with HIV first, pointing to the importance of including the treatment of latent TB infection in the newly HIV diagnosed, in order to minimize the burden of TB and to reduce the risk of death. Among those who had both diagnoses at almost the same time, staring ART can be a crucial to survival. Prevention and control of the HIV and TB epidemics necessarily include coordinate actions to reduce HIV and TB transmission, by encouraging HIV testing among TB cases and referral to ART for those found HIV-infected, and by promoting LTBI treatment among PLHIV, thereby reducing the burden of TB.
